# Supplementary material for: Early Identification of Sepsis-Induced Acute Kidney Injury by Using Monocyte Distribution Width, Red-Blood-Cell Distribution, and Neutrophil-to-Lymphocyte Ratio
Source: Diagnostics (Basel). 2024 Apr 28;14(9):918. doi: 10.3390/diagnostics14090918 (PMC11083534; doi:10.3390/diagnostics14090918)
Supplement: Supplementary file 1 [file diagnostics-14-00918-s001.zip › diagnostics-2941210-supplementary.pdf]

Supplemental Table S1. Shapiro-Wilk Test

Variables	Statistic	df	*P* value
Age	0.965	271	<0.001
MDW	0.903	271	<0.001
RDW	0.732	271	<0.001
initial SOFA	0.855	271	<0.001
MAP	0.967	271	<0.001
NLR	0.719	270	<0.001

Abbreviations: df: degree of freedom; MDW, monocyte distribution width; RDW, red blood cell volume distribution width; SOFA, sepsis-related organ failure assessment; MAP, mean arterial pressure; NLR, neutrophil-to-lymphocyte ratio.

*Statistical significance was defined as $p < 0.05$.
